# Disorders of the Optic Nerve in Mitochondrial Cytopathies: New Ideas on Pathogenesis and Therapeutic Targets

**DOI:** 10.1007/s11910-012-0260-0

**Published:** 2012-03-04

**Authors:** Kamil S. Sitarz, Patrick F. Chinnery, Patrick Yu-Wai-Man

**Affiliations:** 1 Wellcome Trust Centre for Mitochondrial Research, Institute of Genetic Medicine, Newcastle University, Newcastle, UK; 2 Department of Ophthalmology, Royal Victoria Infirmary, Newcastle upon Tyne, UK; 3 Wellcome Trust Centre for Mitochondrial Research, Institute of Genetic Medicine, Centre for Life, Newcastle University, Newcastle upon Tyne, NE1 3BZ UK

**Keywords:** Dominant optic atrophy, Haplogroup, Hereditary spastic paraplegia, Heteroplasmy, Idebenone, Leber hereditary optic neuropathy, Mitochondrial DNA, Mitofusin, Multiple sclerosis, Neuroprotection, Retinal ganglion cell, Optic nerve, Mitochondrial cytopathies

## Abstract

Mitochondrial cytopathies are a heterogeneous group of human disorders triggered by disturbed mitochondrial function. This can be due to primary mitochondrial DNA mutations or nuclear defects affecting key components of the mitochondrial machinery. Optic neuropathy is a frequent disease manifestation and the degree of visual failure can be profound, with a severe impact on the patient’s quality of life. This review focuses on the major mitochondrial disorders exhibiting optic nerve involvement, either as the defining clinical feature or as an additional component of a more extensive phenotype. Over the past decade, significant progress has been achieved in our basic understanding of Leber hereditary optic neuropathy and autosomal-dominant optic atrophy—the two classical paradigms for these mitochondrial optic neuropathies. There are currently limited treatments for these blinding ocular disorders and, ultimately, the aim is to translate these major advances into tangible benefits for patients and their families.

## Introduction

**Figure 1:**
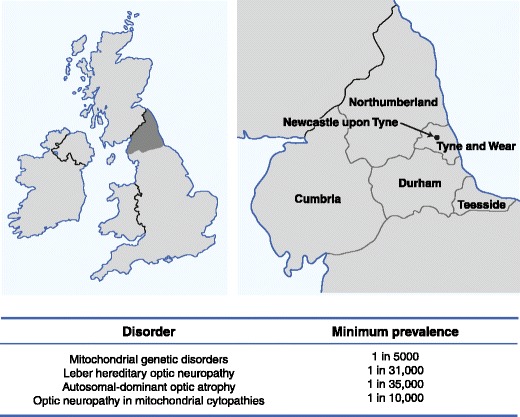
Mitochondrial disease in the North of England. (Adapted from Yu-Wai-Man et al. [2] and Schaefer et al. [50])

Mitochondria are found in all nucleated cells and reflecting this ubiquitous presence, patients with mitochondrial cytopathies often manifest a diverse combination of tissue and organ involvement [1]. However, for reasons that still remain unclear, mitochondrial dysfunction has a marked predilection for the optic nerve, the latter being affected in about half of all patients with confirmed mitochondrial disease (Fig. 1). Irrespective of the molecular pathways involved, remarkably, these mitochondrial optic neuropathies all share the same pathological features—selective degeneration of the retinal ganglion cell (RGC) layer, leading to progressive axonal loss and the onset of visual failure [2]. In this review, recent advances in our understanding of this important group of disorders are discussed, in addition to promising therapeutic strategies.

## Leber Hereditary Optic Neuropathy

### Epidemiology

Leber hereditary optic neuropathy (LHON, OMIM 535000) is named after Theodore Leber (1840–1917), a German ophthalmologist who was the first to describe the key features of this disorder [3]. LHON is the most common of the primary mitochondrial DNA (mtDNA) disorders and the minimum prevalence has been estimated at 1 in 31,000 in the North of England (Fig. 1) [4]. Epidemiological studies from the Netherlands and Finland have reported comparable figures of 1 in 39,000 and 1 in 50,000, respectively. In one Australian study, 2% of individuals on the National Blind Registry had optic atrophy secondary to LHON, highlighting the significant socioeconomic burden of this inherited optic nerve disorder. Three mtDNA point mutations—m.3460 G > A, m.11778 G > A, and m.14484 T > C—account for the vast majority (~ 90%) of LHON cases and, for this reason, they are often referred to as “primary” (Table 1) [3, 5].

**Table 1:** mtDNA variants associated with LHON

	Mitochondrial gene	Nucleotide change
Common variants (~ 90%)	*MTND1*	m.3460 G > A^a^
	*MTND4*	m.11778 G > A^a^
	*MTND6*	m.14484 T > C^a^
Rare variants (~ 10%)	*MTND1*	m.3376 G > A, m.3635 G > A^a^, m.3697 G > A, m.3700 G > A, m.3733 G > A^a^, m.4025 C > T, m.4160 T > C, m.4171 C > A^a^
	*MTND2*	m.4640 C > A, m.5244 G > A
	*MTND3*	m.10237 T > C
	*MTND4*	m.11696 G > A, m.11253 T > C
	*MTND4L*	m.10663 T > C^a^
	*MTND5*	m.12811 T > C, m.12848 C > T, m.13637 A > G, m.13730 G > A
	*MTND6*	m.14325 T > C, m.14568 C > T, m.14459 G > A^a^, m.14729 G > A, m.14482 C > A^a^, m.14482 C > G^a^, m.14495 A > G^a^, m.14498 C > T, m.14568 C > T^a^, m.14596 A > T
	*MTATP6*	m.9101 T > C
	*MTCO3*	m.9804 G > A
	*MTCYB*	m.14831 G > A

Over 70% of LHON carriers harbor the m.11778 G > A mutation, but as a result of a founder event, the m.14484 T > C mutation has been identified in nearly 90% of all affected patients of French Canadian descent [3, 5]. In most laboratories worldwide, the diagnostic protocol involves screening for the three “primary” LHON mutations in the first instance. As full mitochondrial genome sequencing remains time consuming and expensive, this is only indicated if the initial LHON screen is negative and there is a strong clinical suspicion

^a^These mtDNA variants are definitely pathogenic. They have been identified in two or more independent LHON pedigrees, showing segregation with affected disease status. The remaining putative LHON mutations have been found in singleton cases or in a single family, and additional evidence is required before pathogenicity can be irrefutably ascribed

*LHON* Leber hereditary optic neuropathy; *mtDNA* mitochondrial DNA

### Clinical Manifestations

Typically, disease conversion in LHON is characterized by acute or subacute central visual loss in one eye, followed 2 to 4 months later by the fellow eye [3, 5]. Unilateral optic nerve involvement in LHON is exceptionally rare and another underlying pathological process should be actively excluded in these atypical cases. Bilateral simultaneous onset probably occurs in about 25% of patients, although it can be difficult for some individuals to accurately report whether visual loss had been ongoing in one eye prior to the fellow eye being affected. The peak age of onset is in the second and third decades of life and it is unusual for symptoms to develop beyond 50 years of age.

The initial visual loss in LHON is severe and it usually plateaus over the next 6 months, with most patients achieving visual acuities of 20/200 or worse [3, 5]. There is an associated dense central or centrocecal scotoma and color vision is significantly impaired. The pupillary light reflexes are relatively preserved and this rather surprising feature has recently been ascribed to a special class of melanopsin-containing RGCs that are more resistant to the mtDNA LHON mutations [6••]. In the acute phase, classically, there is optic disc hyperemia, peripapillary telangiectatic vessels, vascular tortuosity, and retinal nerve fiber layer (RNFL) edema secondary to axonal stasis. In a proportion (20% to 40%) of LHON carriers undergoing disease conversion, the optic discs can look entirely normal, followed 6 weeks later by the development of neuroretinal rim pallor [5]. Pathological cupping of the optic disc is also a recognized feature of longstanding LHON cases, reflecting the ongoing loss of RGC axons in the chronic phase of the disease. The overall prognosis in LHON is poor, even among patients harboring the m.14484 T > C mutation, which carries the best chance for partial visual recovery [5]. If it occurs, a slow improvement in visual parameters can be expected within the first year, although more delayed visual recovery has been reported.

### Syndromal LHON Phenotypes

LHON is typically a monosymptomatic disease but additional features such as cardiac conduction defects, peripheral neuropathy, dystonia, and myopathy have been reported as occurring more frequently among LHON carriers. In particular, there is a well-reported association between the three primary mtDNA LHON mutations and a multiple sclerosis–like illness, especially among female carriers. Rarer pathogenic mtDNA variants have been linked with more atypical “LHON +” syndromes, where the optic neuropathy segregated with prominent neurological features including spastic dystonia, ataxia, juvenile-onset encephalopathy, and psychiatric disturbances [2].

### Disease Modifiers

LHON is characterized by incomplete penetrance and a marked sex bias, with only about 50% of male carriers and about 10% of female carriers losing vision during their lifetime. The pathological consequences of the mtDNA LHON mutations are clearly being influenced by other disease modifiers, and these can be classified into four main groups: 1) the mitochondrial genetic background, 2) nuclear susceptibility genes, 3) hormonal differences, and 4) environmental triggers.

As mitochondria have a high copy number genome, LHON carriers can be homoplasmic (100% mutant) for the mtDNA mutation, or heteroplasmic with a combination of both the wild-type and mutant mtDNA species. Although the risk of visual loss is minimal at heteroplasmy levels below 60%, this observation cannot account for the bulk of unaffected LHON carriers, most of whom are homoplasmic mutant and therefore beyond this biochemical threshold [7]. MtDNA is highly polymorphic and during human evolution, ancient mtDNA variants have clustered together in specific combinations known as haplogroups. As mtDNA haplogroups influence mitochondrial oxidative phosphorylation, these more subtle variations could magnify or lessen the impact of the LHON mutation on RGC survival. In a large study involving 159 Caucasian LHON pedigrees, haplogroup J was associated with a significantly increased risk of visual loss among m.11778 G > A and m.14484 T > C carriers, whereas m.3460 G > A carriers were more likely to become affected on a haplogroup K background [8]. Haplogroup H had a protective effect but only among individuals harboring the m.11778 G > A mutation. Haplogroup associations have been reported in other ethnic groups, supporting a contributory role for the mtDNA background in influencing LHON penetrance [9].

The predilection for males to lose vision in LHON cannot be explained by mitochondrial genetics and a visual loss susceptibility gene on the X chromosome has long been suspected, the so-called two-locus disease model [10]. Although such an X-linked disease modifier gene has yet to be formally identified, three independent linkage studies have revealed overlapping candidate regions, providing strong evidence for its existence [11–13]. Another obvious factor to account for the observed male bias in LHON is a protective influence conferred by female sex hormones. This hypothesis was recently investigated using LHON cybrid cell lines, and interestingly, treatment with 17β-estradiol resulted in reduced reactive oxygen species (ROS) levels, increased activity of the antioxidant enzyme superoxide dismutase, and more efficient mitochondrial biogenesis [14•].

Various environmental factors have been implicated in precipitating visual loss among LHON carriers including head trauma, industrial toxins, and drugs with mitochondrial toxic effects [15]. The evidence for these environmental triggers is largely anecdotal, but a recent multicenter study of 125 LHON pedigrees has provided convincing evidence for an increased risk of visual failure among smokers, and to a lesser extent, heavy drinkers [16•].

## Mitochondrial Encephalomyopathies

The mitochondrial encephalomyopathies encompass several distinct phenotypes such as mitochondrial encephalomyopathy, lactic acidosis, and stroke-like episodes (MELAS), myoclonic epilepsy and ragged-red fibers (MERRF), maternally inherited Leigh syndrome (MILS), mitochondrial neurogastrointestinal encephalomyopathy (MNGIE), and the Kearns-Sayre syndrome (KSS) [1, 17]. Although variable and not a disease-defining feature, the occurrence of optic atrophy is well described in this group of patients, exacerbating the already considerable disease burden [18]. Additional studies are required to determine the true prevalence of both clinical and subclinical optic neuropathy in these mitochondrial encephalomyopathies, and whether specific mtDNA mutations or phenotypes are linked with an increased risk of optic nerve involvement.

## Nuclear Mitochondrial Disorders

### Autosomal-Dominant Optic Atrophy

#### Epidemiology

Frederick Batten (1865–1918), a pediatric neurologist, is credited with the first report of autosomal-dominant optic atrophy (DOA) in one British family. The characteristic features were described in greater detail by Poul Kjer in a large cohort of Danish families, establishing DOA as a distinct clinical entity from LHON [3]. The prevalence of DOA was estimated at 1 in 35,000 in the North of England (Fig. 1), but additional families have since been identified and the true figure is likely to be much higher (Yu-Wai-Man, Unpublished data).

#### Disease Genes and Candidate Loci

Between 50% and 60% of families with DOA harbor pathogenic mutations in *OPA1*, which codes for a mitochondrial inner membrane protein (Table 2) [3, 5]. *OPA1* is highly polymorphic and over 200 pathogenic variants have been identified, clustering in the GTPase region and the dynamic central domain. To minimize cost, *OPA1* is routinely sequenced using polymerase chain reaction–based methods, which will not detect exonic deletions or duplications. These large-scale rearrangements have been identified in 10% to 20% of *OPA1*-negative families, and these additional molecular studies, although not yet widely available, are recommended for probands with a clear-cut DOA phenotype and a strong family history [2].

**Table 2:** Nuclear mitochondrial disorders with prominent optic nerve involvement

Inheritance	Locus	Gene	OMIM	Phenotypes
Dominant	1p36.2	*MFN2*	601152	Hereditary motor and sensory neuropathy type 6 (HMSN-6, CMT2A)
	3q28–q29	*OPA1*	165500	Isolated optic atrophy and syndromal dominant optic atrophy (DOA+)
	19q13.2–q13.3	*OPA3*	165300	Autosomal-dominant optic atrophy and early-onset cataracts (ADOAC)
Recessive	9q13–q21.1	*FXN*	229300	Friedreich’s ataxia (FRDA)
	11q14.1–q21	*TMEM126A*	612989	Optic atrophy ± auditory neuropathy
	16q24.3	*SPG7*	607259	Hereditary spastic paraplegia type 7 (HSP-7)
	19q13.2–q13.3	*OPA3*	258501	Type III 3-methylglutaconic aciduria (Costeff syndrome)

(Adapted from Yu-Wai-Man et al. [2, 5])

Mutations in *OPA3* were first described in Iraqi-Jewish families with autosomal-recessive type III 3-methylglutaconic aciduria (Costeff syndrome), a progressive neurodegenerative disorder with early-onset optic atrophy, hypotonia, ataxia, extrapyramidal dysfunction, and cognitive decline [19]. Heterozygous *OPA3* mutations were subsequently reported in two French families with a dominantly inherited form of optic atrophy associated with premature cataract formation (ADOAC) [20]. Similar to *OPA1*, *OPA3* has a mitochondrial-targeting domain and it was initially considered to be a mitochondrial inner membrane protein. However, a recent study has suggested instead that *OPA3* localizes to the mitochondrial outer membrane, with its C-terminus facing the cytosol [21]. The causative genetic defects in the remaining families with DOA have not yet been identified, although a number of candidate loci have been reported (Table 2).

#### Visual Function and Disease Progression

Visual loss in DOA is bilateral and symmetrical with the majority patients reporting subnormal vision from early childhood. There is a wide intra- and inter-familial variability in disease severity, with visual acuities ranging from 20/20 to the detection of hand movement [2, 3]. Individuals with visual acuities of 20/30 or better are frequently asymptomatic and they are only identified as having features of a bilateral optic neuropathy at the time of contact tracing. On long-term follow-up, visual function was observed to deteriorate in 50% to 75% of patients with DOA but the rate of progression was highly variable, making genetic counseling difficult [2, 3]. Although a milder optic nerve disease compared with LHON, DOA still results in significant visual impairment with nearly half of all patients eventually registered blind.

As in LHON, the primary site of pathology in DOA is the papillomacular bundle and central, centrocecal, and paracentral scotomas are the most common visual field defects. The degree of dyschromatopsia is commensurate with the level of vision and pure tritanopia, once considered a pathognomonic feature of DOA, is only rarely seen. Histopathological studies of a postmortem eye retrieved from one patient showed relative preservation of melanopsin-containing RGCs, accounting for the lack of an afferent pupillary defect in DOA—another peculiarity shared with LHON [6••]. The optic nerve head in DOA can look diffusely pale or it can have a characteristic temporal wedge, especially in patients with early disease where RGC loss remains mostly localized to the papillomacular bundle [2, 3].

#### The Expanding Phenotypic Spectrum

Up to 20% of patients harboring *OPA1* mutations will develop a syndromal form of the disease (DOA+) marked by significant neurological complications [22•]. The most common extraocular feature observed in this group is sensorineural hearing loss, which usually manifests itself in the second and third decades of life after visual failure has become apparent. A proportion of *OPA1* carriers will progress to a more debilitated state with variable combinations of ataxia, peripheral neuropathy, myopathy, and progressive external ophthalmoplegia in later life. From a mechanistic perspective, it is also revealing that some patients with DOA+ can present with clinical features indistinguishable from other neurodegenerative disorders such as multiple sclerosis, hereditary spastic paraplegia (HSP), and the inherited spinocerebellar degenerations. The phenotypic spectrum of *OPA1* disease is likely to expand even further with greater clinical awareness and easier access to molecular genetic testing.

#### Other Nuclear Mitochondrial Optic Neuropathies

The nuclear defects underlying several common neuromuscular disorders have been clarified. A remarkable element has been the increasing realization that mitochondrial dysfunction plays a central role in the pathophysiology of disease groups as diverse as Charcot-Marie-Tooth (CMT) disease, HSP, and the inherited spinocerebellar ataxias—reflecting to a certain extent the broader phenotypic spectrum recently described for *OPA1* disease [2]. Mutations in *MFN2*, which codes for a critical mitochondrial outer membrane protein, have been identified in patients with hereditary motor and sensory neuropathy type 6 (HMSN-6), an autosomal-dominant axonal CMT subtype (Table 2) [23]. Affected individuals develop an early-onset peripheral neuropathy with a progressive bilateral optic atrophy in later childhood. Optic neuropathy also features prominently in Friedreich ataxia and HSP type 7 (HSP-7), caused by nuclear mutations in *FXN* and *SPG7*, respectively [2]. Frataxin is essential for the assembly of the iron-sulfur clusters embedded within the mitochondrial respiratory chain complexes, whereas paraplegin is a key mitochondrial protease involved in the proteolytic processing of *OPA1* [24, 25]. All these disease pathways are likely interrelated, and disturbance in one element will set into motion a vicious cycle, which eventually disturbs mitochondrial homeostasis triggering neuronal cell loss. As the molecular basis of other inherited human diseases is uncovered, it will be interesting to note whether optic atrophy is present in those cases where the genetic defect is ultimately revealed to affect mitochondrial function. A recent example is the identification of *TMEM126A* mutations among patients with autosomal-recessive optic atrophy, with or without an associated auditory neuropathy [26]. Although its exact functions still remain to be determined, fascinatingly, TMEM126A is a mitochondrial transmembrane protein present at high levels within the RGC layer and the optic nerve head [26].

## Mechanisms Contributing to RGC Loss

The final pathological outcome in mitochondrial optic neuropathies is apoptotic RGC loss and several disease pathways can contribute to this irreversible process. Although a greater emphasis has been placed on the experimental data obtained for LHON and DOA, similar mechanisms have also been shown to operate in other mitochondrial cytopathies.

### Bioenergetic Failure

Mitochondria are the cell’s powerhouses providing most of its adenosine triphosphate (ATP) requirements through the tight control of mitochondrial respiratory chain activity. All three primary LHON mutations—m.3460 G > A, m.11778 G > A, and m.14484 T > C—disrupt key polypeptide subunits of complex I. Similarly, *OPA1* mutations have a detrimental impact on mitochondrial oxidative output by impairing the assembly and stability of the respiratory chain complexes [27]. Most studies, based on in vitro or in vivo assays, indicate a significant impairment in complex I activity, leading to a reduction in mitochondrial membrane potential and overall ATP synthesis (reviewed in [5] and [15]). Although this bioenergetic deficit is bound to impact negatively on RGCs, by itself, this cannot account for their selective vulnerability. Photoreceptors, for example, have a much higher energetic demand and outer retinal function is usually preserved in both LHON and DOA.

### ROS and Excitotoxicity

The disrupted flow of high-energy electrons along the respiratory chain leads to free radical production and increased ROS levels have been consistently observed in transmitochondrial LHON cybrids (reviewed in [5] and [15]). The efficient reuptake of glutamate is also disrupted in these cellular models due to the downregulation of excitatory amino acid transporter (EAAT1) activity. A *drosophila Opa1* (*dOpa1*) model has recently been established and homozygous mutant flies developed a rough and glossy eye phenotype due to the loss of hexagonal lattice cells, with decreased lens and pigment deposition. This genetically engineered *dOpa1* mutation led to a dramatic increase in ROS levels, which could be partially rescued with dietary antioxidant supplementation or overexpression of *SOD1* [28]. Heterozygous adult flies were phenotypically normal but similar to the homozygotes, ROS levels were elevated with clear evidence of cellular oxidative stress. Increased ROS levels and glutamate excitotoxicity clearly represent a toxic combination, sufficiently potent to initiate the apoptotic cascade.

### Disturbed Calcium Handling

Besides its localization to the mitochondrial outer membrane, MFN2 is also associated with the endoplasmic reticulum (ER), resulting in a close communication between the mitochondrial and ER membranes [29••, 30]. When the MFN2 protein is disrupted or absent, as in CMT-2A this tethering effect is lost with dramatic consequences on cellular calcium homeostasis. The ER and mitochondrial network are important calcium stores and the dynamic flux between these two compartments buffers against cytosolic calcium spikes, which can sensitize the cell to various pro-apoptotic signals such as glutamate [29••, 30]. Mirroring the pathomechanism of *MFN2* mutations, mtDNA point mutations involving complex I and complex IV subunits have also been directly linked with disturbed calcium handling in neuronal populations [31]. Although speculative, it is likely that a similar process is operating in LHON and DOA, contributing to RGC dysfunction and ultimately disease conversion among at-risk mutational carriers [32]. The emerging links between calcium homeostasis and mitochondrial dysfunction are an exciting new development, highlighting potential disease pathways amenable to therapeutic intervention.

### Mitochondrial Network Dynamics

Cytochrome *c* is a powerful pro-apoptotic mediator and as a protective mechanism, high concentrations of these molecules are carefully sequestered within the mitochondrial cristae. Irrespective of the mtDNA mutation involved, the dissipation of the mitochondrial membrane potential accelerates the cytosolic release of these cytochrome *c* molecules, precipitating an irreversible commitment toward programmed cell death [33]. The identification of *OPA1* and *MFN2* mutations in DOA and CMT-2A has provided further insights into this key disease mechanism. OPA1 ensures the integrity of the mitochondrial cristae’s tight junctions, preventing cytochrome *c* from leaching out into the cytosolic space. In addition, OPA1 and MFN2 both belong to the dynamin superfamily of mechanoenzymes, sharing considerable structural similarities, including a highly conserved catalytic GTPase domain [24]. These two proteins have important pro-fusional properties and by working closely together, they maintain a highly-interconnected mitochondrial network throughout the cell’s structure [24, 33]. Unsurprisingly, the pathological hallmark of *OPA1*- and *MFN2*-mutant fibroblasts is mitochondrial network fragmentation, which not only impairs mitochondrial oxidative phosphorylation, but also leads to the uncontrolled release of calcium and cytochrome *c* into an already compromised cellular environment [27, 34, 35••].

### Somatic mtDNA Defects

Pathological levels of cytochrome *c* oxidase–negative fibers have been identified in skeletal muscle biopsies from patients with DOA+ [35••, 36••]. The underlying biochemical defect in these muscle fibers is secondary to the accumulation of high levels of somatic mtDNA deletions, which have clonally expanded during the patient’s lifetime, partly accounting for the delayed onset of these extraocular features [37••]. Intriguingly, the risk of developing DOA+ is three times higher with missense mutations targeting the functional GTPase domain, consistent with a dominant-negative mutational effect [22•]. OPA1 is clearly involved in preserving the integrity of the mitochondrial genome and recent studies have provided some tantalizing insights into the mechanisms that contribute to mtDNA instability [38], [39••, 40••]. OPA1 is thought to anchor the mtDNA replicative machinery, known as nucleoids, to the mitochondrial inner membrane and a dominant-negative mutation could plausibly upset this delicate balance, leading to mtDNA deletion formation [40••]. Why is this relevant to our understanding of RGC loss? Patients with DOA+ have worse visual acuities and significantly thinner RNFL thickness compared with those who only develop isolated optic atrophy [41]. The accumulation of these somatic mtDNA deletions is therefore clearly having an incremental effect on the other deleterious consequences linked to *OPA1* mutations.

## Why Are RGCs Selectively Vulnerable?

One factor that could explain this tissue-specific vulnerability is the rather unusual anatomical peculiarity encountered at the lamina cribrosa, where RGC axons first acquire their myelin sheaths. This transition is marked by a sharp differential gradient, with a much higher density of mitochondria and voltage-gated sodium channels in the pre-laminar unmyelinated segment of the optic nerve [42, 43]. These physiological adaptations facilitate the efficient propagation of action potentials in the absence of an insulating myelin covering. The pre-laminar region therefore represents a weak link, significantly more exposed to the disadvantageous consequences of, even subtle, mitochondrial biochemical defects.

Histopathological studies of optic nerves retrieved from two patients with LHON, one harboring the m.3460 G > A mutation and the other the m.11778 G > A mutation, have revealed some interesting observations on the susceptibility of specific RGC populations (reviewed in [2] and [15]). A prominent loss of smaller-caliber axons was observed, corresponding to the parvocellular RGC population, whereas the larger-caliber magnocellular RGCs were relatively preserved. These ultrastructural findings were subsequently confirmed with higher-resolution transmission electron microscopy. Parvocellular RGCs are a major component of the papillomacular bundle and this greater vulnerability to impaired mitochondrial oxidative phosphorylation could be related to their relatively smaller cross-sectional areas, the latter further exacerbating axonal stasis in conditions of sustained metabolic stress.

The maintenance of a higher mitochondrial concentration in the pre-laminar region also highlights the central role played by the cytoskeleton, especially the microtubule network, in channeling mitochondria to their appropriate cellular locations. Axonal transport in highly specialized neuronal populations such as RGCs is critically dependent on these mitochondrial-cytoskeletal interactions (reviewed in [2] and [15]). These can be adversely affected either by an underlying mitochondrial respiratory chain defect, or by a primary disturbance in microtubule assembly—the pathological hallmark of the HSP group of disorders [2]. Optic atrophy is well described in HSP-7 and it is likely that optic nerve involvement remains an under-reported feature in other HSP genetic subtypes.

## Therapeutic Interventions

### Visual Rehabilitation

The majority of patients with mitochondrial optic neuropathies are severely visually impaired and the sudden onset of visual loss in otherwise healthy individuals carries a significant psychological and socioeconomic burden. Therefore, clinicians have an important role to play in facilitating access to rehabilitative services such as low visual aids and occupational therapy.

### Clinical Surveillance

It is essential to screen for associated systemic complications, such as diabetes and cardiomyopathy, among patients with mitochondrial cytopathies. The development of diabetic peripheral neuropathy and cardiac-related exertional dyspnea can further compound the physical difficulties faced by patients with poor vision, and they should be aggressively managed as part of a multidisciplinary team. As a general health measure, patients with mitochondrial disease should be advised not to smoke and to moderate their alcohol intake. Furthermore, in LHON, smoking, and to a lesser extent excessive binge drinking, have been linked with an increased risk of visual loss [16•].

### Pharmacological Agents

Various pharmacological agents with putative neuroprotective properties have been used to mitigate the deleterious consequences of mitochondrial dysfunction and to prevent further RGC loss (reviewed in [44••]). The most promising agent to date is idebenone (2,3-dimethoxy-5-methyl-6-[10-hydroxydecyl]-1,4-benzoquinone), a short-chain synthetic benzoquinone analogue, which promotes mitochondrial ATP synthesis in addition to having antioxidant properties. Coenzyme Q10 (CoQ_10_) is a longer-chain quinone analogue and based on limited evidence, CoQ_10_ supplementation is often used to treat patients with mitochondrial cytopathies. Idebenone is thought to have some advantageous properties over CoQ_10_, both in terms of its bioavailability and its mode of action. Unlike CoQ_10_, idebenone is able to bypass complex I inhibition by shuttling electrons directly from the cytosol to complex III, thereby restoring ATP production and decreasing lactate levels [45].

The results of a multicenter randomized placebo-controlled trial (RHODOS [Rescue of Hereditary Optic Disease Outpatient Study]) of idebenone in LHON have recently been released. Idebenone was well tolerated at a dose of 900 mg per day and no adverse drug-related events were reported. The visual outcome data indicate that patients with discordant visual acuities (LogMAR > 0.2), and thus at highest risk of further deterioration in the least affected eye, were more likely to benefit from treatment with idebenone [44••]. The findings from the RHODOS trial are promising and for the first time, they offer the hope of preserving vision for affected LHON carriers who are still at an early stage of the disease process.

### Looking into the Future: Gene Therapy

Gene therapy for primary mitochondrial cytopathies poses several discrete challenges; the mitochondrial inner membrane is a relatively impermeable membrane that needs to be bypassed, and a highly efficient vector is needed to transfect a sufficient number of mitochondria per cell to achieve the desired effect. A possible solution is to bypass the mitochondrial genome using an allotopic approach. Instead, the gene of interest is transfected into the nuclear genome and the protein product is engineered with a specific targeting sequence that facilitates its uptake into the mitochondrial compartment, thereby compensating for the mtDNA mutation. Proof of principle has been demonstrated in experimental LHON models where adeno-associated virus vectors have been used to successfully transfect cells with a replacement wild-type mtDNA allele or neuroprotective genes such as *SOD2* [46–48]. These results are encouraging and allotopic rescue could be easily applied to the RGC layer which is easily accessible. However, key issues of safety and efficacy need to be further addressed before their application to human clinical trials can be advocated [2].

### Preventing Disease Transmission

Several strategies are currently being explored to prevent the transmission of pathogenic mitochondrial and nuclear mutations among women of child-bearing age. The difficult technical and ethical issues raised by preimplantation genetic testing and new in vitro fertilization techniques such as pronuclear transfer are outside the scope of this review, and these have been detailed elsewhere [2, 49].

## Conclusions

A number of recurring disease mechanisms have been identified that contribute to RGC loss in mitochondrial optic neuropathies. These provide a unique opportunity for targeted therapeutic interventions aimed not only at improving visual function, but also the neurological deficits seen in the more severe mitochondrial cytopathies. However, despite these major advances, the risk factors underpinning the selective vulnerability of RGCs have yet to be clarified. Research in this area has been severely limited by the lack of diseased human optic nerves but hopefully, with the development of faithful animal models and more advanced biotechnological tools, we will soon be in a position to disentangle these fundamental research questions both at the structural and molecular levels.
